# Physician Use of Large Language Models: A Quantitative Study Based on Large-Scale Query-Level Data

**DOI:** 10.2196/76941

**Published:** 2025-08-25

**Authors:** Lin Qiu, Chuang Tang, Xuan Bi, Gordon Burtch, Yanmin Chen, Heping Zhang

**Affiliations:** 1 Department of Information Systems and Management Engineering, Southern University of Science and Technology, CoE North 907, 1088 Xueyuan Ave, Shenzhen, 518055, China, 86 0755 88012425; 2 Peking University HSBC Business School, Shenzhen, China; 3 Department of Information and Decision Sciences, Carlson School of Management, University of Minnesota, Minnesota, MN, United States; 4 Department of Information Systems, Questrom School of Business, Boston University, Boston, MA, United States; 5 Shenzhen Maternity and Child Healthcare Hospital, Southern Medical University, Shenzhen, China; 6 Department of Biostatistics, Yale University School of Public Health, New Haven, CT, United States

**Keywords:** generative AI, large language models, health care, generative AI usage, privacy, artificial intelligence, generative artificial intelligence

## Abstract

**Background:**

Generative artificial intelligence (GenAI) has rapidly emerged as a promising tool in health care. Despite its growing adoption, how physicians make use of it in medical practice has not been qualitatively studied. Existing literature has largely focused on theoretical applications or experimental validations, with limited insight into real-world physician engagement with GenAI technologies.

**Objective:**

The aim of this study was to leverage a fine-grained dataset at the query level to quantitatively examine how physicians incorporate GenAI into their clinical and research workflows. The primary objective was to analyze usage patterns over time and across physician demographics. A secondary goal was to assess potential risks to patient privacy arising from physicians’ interactions with GenAI platforms.

**Methods:**

This study collected 106,942 query-and-answer pairs by 989 physicians between August 29, 2023, and April 16, 2024. We performed topic classification to identify the most prevalent use cases, examining how these use cases evolved over time and across demographics. We also developed sensitivity classifiers to detect personally identifiable information in physicians’ queries to explore the potential privacy breach risks around physicians’ use of GenAI.

**Results:**

Approximately 40% (396/989) of the enrolled physicians were female, 45.9% (454/989) were younger than 25 years, and 54.1% (535/989) were between 25 and 56 years of age. The majority of them worked in clinical departments (680/989, 68.8%) or medical technology departments (127/989, 12.8%). Our classification-based quantitative analyses suggest the following. First, physicians use GenAI predominantly for medical research (64,379/106,942, 60.2%) rather than clinical practice (13,100/106,942, 12.25%). Second, physicians focus more on health care–related questions (rising from 64,165/106,942, 60% to 83,415/106,942, 78%) within the first 15% (16,041/106,942) of their query sequence. Third, the use of GenAI differed across physician demographics and features. Specifically, female physicians asked a larger proportion of clinical questions (female: 0.154 vs male: 0.108; *P*<.001) and administration questions (female: 0.027 vs male: 0.018; *P*<.001) than male physicians; younger physicians posed more clinical questions (age ≤25: 0.146 vs age ∈ (25, 40]: 0.115 vs age >40: 0.103; *P*<.001) but fewer research questions (age ≤25: 0.580 vs age ∈ (25, 40]: 0.607 vs age >40: 0.664; *P*<.001) than senior physicians; and physicians accessing GenAI via computers asked more research questions (computer: 0.637 vs mobile: 0.296; *P<.*001), whereas physicians using mobile devices asked more clinical questions (computer: 0.107 vs mobile: 0.264; *P*<.001). Fourth, only 2.68% (2866/106,942) of physician queries contained sensitive information, the majority of which were primarily derived from writing and editing.

**Conclusions:**

Physicians are actively integrating GenAI into their professional routines, primarily leveraging it for research but also increasingly for clinical support. Usage patterns vary significantly across demographic lines, including gender, age, and device preference. Despite the presence of sensitive information in some queries, the risk of privacy breaches appears to be low.

## Introduction

Since its launch in late 2022, ChatGPT has been at the forefront of the burgeoning field of generative artificial intelligence (GenAI), sparking immense enthusiasm yet significant concerns within academic and professional communities [1-4].

GenAI models offer significant potential for innovation in the health care domain [5-11]. They have the potential to streamline time-consuming administrative tasks [12], support informed medical decisions through precise and personalized treatment recommendations [6], serve as an interactive encyclopedia for medical education [7], and drive medical research forward more generally [7]. However, the realization of these benefits depends on various factors. To provide value in clinical decision support, these technologies must first be integrated into existing technological systems [7] and their implementation must be coupled with significant modifications to medical workflows and processes [12]. Unfortunately, comprehensively implementing these technologies within the health care sector is made difficult because GenAI technologies present unique challenges [12]. The most notable risks that have been raised to date include data privacy concerns [12-14] (eg, noncompliance with the Health Insurance Portability and Accountability Act [15]), the concern that clinicians may enter patients’ protected health care information within queries, and “hallucination,” whereby LLMs could potentially mislead physicians [6].

As GenAI increasingly penetrates health care practice, it is essential to understand how physicians are adopting and using these technologies. Existing research on this topic generally falls into 3 main streams. The first stream is studying GenAI models for medical applications, as exemplified by various MedGPTs [16-19]. Although these studies demonstrate potential use cases for GenAI, they are typically conducted in experimental settings [16-19]. How physicians engage with these technologies in real-world settings remains poorly understood. The second stream involves small-scale qualitative research, such as interviews or surveys with limited participant pools [20,21]. Although valuable for exploratory insights, these studies offer limited generalizability due to their constrained sample sizes. The third stream consists of review papers that synthesize existing AI-related research to infer potential uses of GenAI in health care [22,23]. However, these reviews often lack detailed, practice-level insights into how GenAI is being integrated into clinical or research workflows. To date, few studies have quantitatively examined how physicians actually use GenAI in routine clinical or research practice.

Moreover, while many studies have highlighted the potential advantages of GenAI in health care [5-9], few address the challenges associated with its use. At this early stage of GenAI adoption, it remains unclear whether digital divides exist across different demographic groups. Digitally disadvantaged users may be less likely to adopt advanced GenAI tools, thereby hindering the widespread integration of these models. Additionally, given that GenAI systems are not yet formally regulated or integrated into mainstream electronic health record systems, privacy risks have not been systematically assessed.

Given the foregoing situation, this paper explored large-scale query-level data to quantitatively explore the use of GenAI by physicians in practice. Specifically, we sought to answer questions including the following: What types of topics do physicians inquire about? How do these topics evolve with increased engagement and vary across physician demographics? How frequently do the queries contain sensitive information?

## Methods

### Study Design

This study used a classification-based quantitative descriptive design, which is well-suited for examining use patterns and uncovering empirical insights into the use of GenAI [24,25]. We applied separate classification models to identify query topics and detect sensitive information—approaches that have demonstrated strong performance in processing large-scale textual data [26-28]. By categorizing the topics of physician queries, we were able to trace usage trends over time and across demographic groups. In parallel, our sensitivity analysis allowed us to evaluate potential privacy risks associated with physicians’ interactions with GenAI platforms.

### Setting, Participants, and Sampling

Our query-and-answer data were collected from a company in Xiamen, China, which hosts a website that allowed physicians to access popular GenAI (eg, ChatGPT-4 and ChatGPT-3.5 from OpenAI) via these models’ application programming interfaces. This company invited physician participants from Dingxiangyuan, a leading medical community in China. As one of the largest web-based platforms for doctors, researchers, and health care professionals in the country, Dingxiangyuan provides a valuable resource for accessing a broad pool of physician users.

As most of these users were from China, they had limited access to cutting-edge GenAI models via non–application programming interface channels during our study period. The data collected are believed to reflect the majority of participating physicians’ use of GenAI during the observation period. Users’ demographic information, including age, gender, department, and device type, was also collected from this company.

### Data Collection

This study collected 106,942 query-and-answer pairs by 989 physicians between August 29, 2023, and April 16, 2024, to examine how the use of GenAI evolved over time and across demographics. Approximately 40% (n=396) of the enrolled physicians were female, 45.9% (n=454) were younger than 25 years, and 54.1% (n=535) were between 25 and 56 years. The majority worked in clinical departments (n=680, 68.8%) or medical technology departments (n=127, 12.8%).

### Data Analysis

We conducted two sets of text analyses. First, we classified the queries to identify the search topics from physician users. We integrated a GPT-4–based classifier with a rule-based method to perform this classification (for method details, see the Topic Classification section of Multimedia Appendix 1). These classified topics allowed us to determine the topics of physicians’ prevalent use cases and to examine if these use cases significantly varied with physicians’ engagement and their demographics.

Second, we performed an additional text-mining analysis to detect potentially sensitive information existing in the queries. We created a detector that incorporates regular expressions, Microsoft’s Presidio [29], and ChatGPT-based detection to thoroughly search for personally identifiable information (for method details, see the Sensitivity Detection section of Multimedia Appendix 1). By analyzing the detected sensitive information, we categorized and quantified the types and frequency of sensitive data present in physicians’ queries. This allowed us to assess the risks of privacy breaches and identify specific instances where breaches may occur.

### Ethical Considerations

We obtained permission from the Xiamen Moniu Investment Company to use their dataset for our secondary data analysis. At the time of data collection, the company obtained informed consent from all participants, and all records were subsequently anonymized to safeguard privacy and confidentiality. Participants received complimentary access to the GenAI tool as compensation.

## Results

### Query Topics

To understand physicians’ objectives when they engage with GenAI models, we conducted text mining on a broad sample of input queries. Table 1 summarizes 15 topics extracted from user queries. They are divided into 4 broad groups in both health care–related and non–health care settings. As depicted, physicians used the GenAI tool not only for health care–related tasks, including clinical practice, medical research, and administrative work, but also for tasks unrelated to health care. The distribution across all 15 topic categories is also detailed in Table 1. In health care–related queries, a significant proportion of the 106,942 physician queries were related to medical research, such as writing and editing (n=24,688, 23.09%), translation (n=19,835, 18.55%), and data analysis and software use (n=17,412, 16.28%). This implies that physicians primarily use GenAI models for research in health care–related settings. Compared with conventional medical research tools like Google Scholar or PubMed, which serve primarily as search engines and databases for retrieving content, GenAI tools actively generate new text and help synthesize information across sources. Notably, physicians are not just consuming information. They are interactively collaborating with GenAI tools to think through research problems in real time (eg, following the chain of thought in solving data analysis problems). All of these advanced capabilities of GenAI are attracting users’ attention.

Further, our findings revealed a seeming reluctance among physicians to engage in queries related to clinical practice. Examples of queries and answers for clinical practice and medical research are presented in Textbox 1. These examples demonstrate that GenAI can provide reasonable answers to both types of questions. However, physicians searched for clinical practice significantly less frequently than they searched for medical research. Physicians sought information about basic medical science and clinical medicine at rates of 6.80% and 3.66%, respectively. Searches for medication information and surgical techniques were markedly lower, at just 0.94% and 0.85%, respectively. There are several potential underlying reasons for this hesitancy. First, physicians are cautious about sharing patient information on the third-party GenAI platform, even anonymously. Without sufficient patient information, queries related to clinical practice become significantly less effective. Second, the conversational nature of GenAI models requires substantial effort to sequentially input a complete patient health profile [30,31]. This is time-consuming and deters the integration of clinical practice with GenAI models. Third, the current capabilities of GenAI are limited in integrating complex medical information, often resulting in clinical ineffectiveness, particularly when multiple conditions must be considered [32].

In queries unrelated to health care, the majority tended to be informal. The largest proportion of these queries pertained to writing and editing tasks (12.86%), including the preparation of documents for non–health care purposes. An additional share of queries involved informal interactions (8.76%), such as searching for recipes, composing poetry, and writing greetings.

**Table 1.** Distribution of 15 topic categories from queries (N=106,942).

Topic category	Values, n (%)
Health care–related	
Writing and editing	24,688 (23.09)
Translation	19,835 (18.55)
Data analysis and software use	17,412 (16.28)
Medical experimental design	2439 (2.28)
Basic medical science	7267 (6.80)
Clinical medicine	3910 (3.66)
Medication information	1007 (0.94)
Surgical techniques	904 (0.85)
Public education	1222 (1.14)
Hospital governance	971 (0.91)
Not related to health care	
Writing and editing	13,753 (12.86)
Other	9366 (8.76)
Data analysis and software use	3013 (2.82)
Relationships	897 (0.84)
Entertainment	258 (0.24)

Textbox 1. **Examples of topic categories.**

**Writing and editing**

Q. How to Determine a PhD Research Topic in Gynecologic Oncology?

A. Determining a PhD research topic is a process that requires careful consideration and thorough investigation. If you are interested in pursuing doctoral research in the field of gynecologic oncology, the following steps and suggestions may be helpful:

Understand the Field: Begin by extensively reading scientific literature related to gynecologic oncology. Familiarize yourself with current research developments, key issues, and unresolved scientific challenges. This will help you build a strong foundational understanding and stay informed about the latest trends in the field.

Find a Supervisor: Look for a mentor with expertise and experience in gynecologic oncology. A qualified advisor can help you choose a suitable research direction and provide professional guidance and support throughout your study.

Identify a Research Question: Based on your interests and the current state of the field, define a specific research question or hypothesis. Your research topic should be academically significant and practically valuable—aiming to fill existing knowledge gaps or address real-world problems.

Design a Research Plan: Develop a detailed research plan based on your research question. This plan should include the research methodology, experimental design, data collection strategies, and analysis methods.

Set Research Objectives and Expected Outcomes: Clearly define your research objectives and establish reasonable expectations for results. This helps maintain focus during your study and provides criteria for evaluating the success of your research.

Submit a Topic Proposal: Once you have finalized your research direction and discussed it with your supervisor, you will need to submit a formal research proposal according to your university or research institution's requirements.

Please note that the above steps are for reference only. The process of defining a PhD topic may vary by institution. It is best to have detailed discussions with your supervisor or relevant academic advisors to receive tailored guidance.

**Translation**

Q. Please translate: ”本研究旨在比较抗菌光动力疗法和次氯酸钠冲洗（根管冲洗的金标准）在抗粪肠球菌感染的治疗效果”

A. “The aim of this study was to compare the effectiveness of antimicrobial photodynamic therapy and sodium hypochlorite irrigation (the gold standard for root canal irrigation) in the treatment of Enterococcus faecalis infection.”

**Data analysis and software use**

Q. In Mendelian randomization data analysis, if the odds ratio (OR) and p-value are known, how can the beta coefficient and standard error (SE) be calculated?

A. In Mendelian randomization data analysis, the odds ratio (OR) is a measure used to quantify the difference in rates between two groups. The p-value is a statistical indicator used to assess whether this difference is significant. If the OR and p-value are known, you can calculate the beta coefficient and standard error (SE) using the following formulas:

1. First, compute the natural logarithm of the OR: ln(OR) = ln(OR value)

2. Then calculate the beta value: beta = ln(OR) / 1.96

3. Calculate the standard error (SE): SE = 1 / (1.96 × √(sample size of case group / sample size of control group))

Notes:

1. 1.96 is a constant used for computing a 95% confidence interval.

2. The case group refers to the treatment or intervention group, while the control group refers to the comparison or untreated group.

3. Sample size refers to the number of observations in each group.

Using these formulas, you can derive the beta and SE from the OR and p-value. These values are essential for further statistical inference and analysis.

Let me know if you'd like a numerical example or code to calculate these!

**Medical experimental design**

A. What Are the Current Mainstream Steps in a GST Pull-Down Assay?

Q. GST pull-down is a commonly used technique to study protein-protein interactions. The basic procedure includes the following steps:

Cloning and Expression of GST Fusion Protein:

The coding sequence of the target protein is fused with the GST (Glutathione S-Transferase) gene. This fusion gene is then cloned into an appropriate host organism (such as E. coli) for expression of the GST-tagged protein.

Purification of GST Fusion Protein:

The expressed GST fusion protein is purified using affinity chromatography. Glutathione agarose or magnetic beads are typically used because GST has a strong affinity for glutathione, allowing it to bind to the beads during purification.

Preparation of Cell Lysate:

The cells that may contain proteins interacting with the GST fusion protein are lysed using appropriate methods (e.g., mechanical disruption, sonication) to release intracellular proteins.

Affinity Precipitation (Pull-Down):

The cell lysate is incubated with the GST fusion protein immobilized on glutathione beads. This allows potential interacting partner proteins to bind to the GST-tagged protein.

Washing and Elution:

Multiple wash steps are performed to remove non-specific binders. Then, proteins specifically bound to the GST fusion protein are eluted by increasing the glutathione concentration.

Analysis:

The eluted proteins are analyzed by SDS-PAGE, followed by silver staining or Western blotting to identify which proteins interact with the GST fusion protein.

These are the standard steps in a GST pull-down assay. Specific protocols may vary depending on the nature of the proteins and experimental conditions. It is essential to maintain rigor and reproducibility in the experimental design to ensure the accuracy and reliability of the results.

**Basic medical science**

Q. What is relationship between iron metabolism and ferroptosis

A. There is a close relationship between iron metabolism and ferroptosis. Iron is an essential element for sustaining life and participates in many biological processes, including oxygen transport, DNA synthesis, and energy metabolism. However, when there is an excess of iron in the body or iron metabolism becomes dysregulated, it can lead to the production of large amounts of reactive oxygen species (ROS). This induces oxidative stress, damaging cell membranes, proteins, lipids, and DNA, ultimately resulting in cell death—a process known as ferroptosis.

Ferroptosis is a newly identified form of programmed cell death, distinct from apoptosis, necrosis, and autophagy. During ferroptosis, excess iron catalyzes the Fenton reaction, producing hydroxyl radicals—highly reactive free radicals that cause oxidative damage to proteins, lipids, and DNA, leading to cell injury and death.

Currently, ferroptosis is a major focus in tumor biology and neurodegenerative disease research. Many types of cancer cells and cells affected by neurodegenerative diseases show a high dependency on iron. Understanding and regulating the process of ferroptosis could provide new therapeutic strategies for treating these diseases.

**Clinical medicine**

Q. Differential diagnosis between sirenomelia and incomplete caudal regression

A. Sirenomelia (also known as Mermaid Syndrome) is a rare congenital developmental disorder characterized by the fusion of the lower limbs, giving the appearance of a mermaid’s tail. In contrast, incomplete caudal regression refers to the underdevelopment or malformation of the caudal (tail) region, which may manifest as missing or abnormal tail structures.

Although sirenomelia and incomplete caudal regression share certain similarities, they are distinct conditions. Sirenomelia primarily involves fusion of the lower limbs, while incomplete caudal regression is centered around abnormalities in the development of the tail region.

In sirenomelia, additional malformations often accompany limb fusion, including abnormalities of the urogenital system and spinal column. In contrast, incomplete caudal regression typically involves developmental defects limited to the tail region and may not be associated with other organ anomalies.

Therefore, in clinical practice, these two conditions can be differentiated and diagnosed based on the specific features of the lower limbs and caudal region.

**Medication information**

Q. Can pregnant women take Azithromycin tablets?

A. The use of azithromycin during pregnancy should be approached with caution. Although some studies suggest that azithromycin is relatively safe for use during pregnancy, there may still be potential risks, especially when used in early pregnancy, as it could slightly increase the risk of certain birth defects.

Therefore, if you are pregnant and need to take azithromycin, it is essential to do so under a doctor's guidance. Your physician will evaluate the risks and benefits based on your specific condition and stage of pregnancy.

In general, the use of any medication during pregnancy—including antibiotics—should be under medical supervision. Self-medicating or purchasing drugs without a prescription is not recommended.

**Surgical techniques**

Q. Can an Attending Physician Perform a Level-III Surgery?

A. Generally speaking, attending physicians typically do not perform level-III surgeries independently, as such procedures are relatively complex and high-risk. These surgeries usually require the guidance of more senior doctors or collaboration with other specialists.

However, the ability to perform surgeries and the classification of surgical levels may vary depending on the hospital's requirements, the physician’s professional experience, and their training background. Some attending physicians may possess advanced skills in specific fields or procedures and may therefore be authorized to perform certain level-III surgeries. This would require their qualifications and experience to be formally recognized and supported by the healthcare institution and relevant professional bodies.

The most accurate and specific information should come from the medical institution in question or the physician’s official credentials. If you have questions about whether a specific procedure can be performed by an attending physician, it is advisable to consult the hospital’s surgical department or the physician directly.

### Usage Pattern

Our study also examined how physicians use GenAI models. For precision, we focused specifically on the 4 broad groups outlined in Table 1.

**Figure 1.**
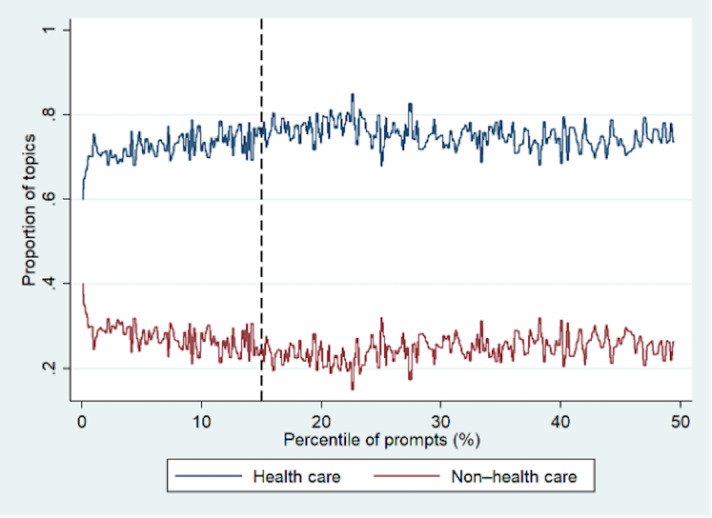
Dynamics of queries. The x-axis represents the percentile of a physician’s queries in sequential order. The y-axis represents the proportion of queries related to either the health care or non–health care topic. The black dashed line indicates a point (approximately the 15th percentile) starting from which the proportion of health care–related queries became relatively high and consistent.

First, we analyzed the temporal dynamics of health care–related (including medical research, clinical practice, and administration groups) versus non–health care topics in user queries, as illustrated in Figure 1. The x-axis represents the percentile of physicians’ queries in sequential order. A higher x-axis value indicates a later query, while a lower value indicates an earlier one. The y-axis represents the proportion of queries related to either the health care or non–health care topic. This plot demonstrates an increase of around 20% in the proportion of health care–related queries and a corresponding decrease of around 20% in non–health care queries within approximately the first 15% of the query sequence, suggesting that engagement with the platform influences the predominance of health care queries. After this “warm-up” period, topic proportions stabilize, indicating a plateau in shifts as user engagement continues. This suggests that physicians start with casual, nonprofessional questions but shift to more serious ones over time, reflecting growing engagement and trust. These trends are also statistically verified through piecewise regression (see the Model Specification section of Multimedia Appendix 1).

Second, we examined the heterogeneity in usage patterns across user demographics. Table 2 displays the group means across different dimensions. First, we observed that the usage patterns among physicians varied by gender. Compared with male physicians, female physicians asked a larger proportion of clinical questions (female: 0.154 vs male: 0.108; *P*<.001) and administration questions (female: 0.027 vs male: 0.018; *P*<.001). Second, age and experience also affected usage. Younger physicians, early in their careers, tended to pose more clinical-related questions (age ≤25: 0.146 vs age ∈ (25,40]: 0.115 vs age >40: 0.103; *P*<.001) compared to their senior counterparts, who were more inclined to inquire about research-related topics (age ≤25: 0.580 vs age ∈ (25,40]: 0.607 vs age >40: 0.664; *P*<.001). This suggests that, unlike younger physicians, experienced physicians seemingly rely more on their own expertise in clinical settings but seek external input more frequently when engaging in research activities. Third, usage patterns varied based on the device used. Physicians accessing the platforms via computers asked more research-oriented questions (computer: 0.637 vs mobile: 0.296; *P*<.001), whereas those using mobile devices primarily inquired about clinical (computer: 0.107 vs mobile: 0.264; *P*<.001) and non–health care (computer: 0.238 vs mobile: 0.393; *P*<.001) topics. This discrepancy indicates that clinical and casual queries often arise spontaneously and are more likely to be addressed on-the-go, while research activities are typically conducted in a more serious manner, for example, via using desktops. Fourth, the department (ie, specialty) of physicians mattered. Among all departments, staff from the clinical department asked the most research questions (clinical department: 0.634 vs medical technology department: 0.540 vs other department: 0.418; *P*<.001) and those from the medical technology department asked the most clinical questions (clinical department: 0.133 vs medical technology department: 0.141 vs other department: 0.078; *P*<.001). We further used regression analyses to examine the heterogeneity in usage patterns across user demographics. The results are shown in Table 3. We found that the heterogeneity in usage patterns remained consistent even after controlling for other demographic factors.

**Table 2.** Average proportions of queries across 4 topic groups.^a^

	Clinical	Research	Administration	Non–health care
Gender				
Female	0.154	0.588	0.027	0.232
Male	0.108	0.609	0.018	0.265
*P* value	<.001	<.001	<.001	<.001
Age (years)				
≤25	0.146	0.58	0.02	0.254
(25,40]	0.115	0.607	0.02	0.258
>40	0.103	0.664	0.024	0.208
*P* value	<.001	<.001	<.001	<.001
Device type				
Computer	0.107	0.637	0.018	0.238
Mobile	0.264	0.296	0.043	0.393
*P* value	<.001	<.001	<.001	<.001
Department				
Clinical	0.133	0.634	0.022	0.211
Medical tech	0.141	0.54	0.027	0.293
Others	0.078	0.418	0.014	0.49
*P* value	<.001	<.001	<.001	<.001

a We evaluated the average proportions of queries across the 4 topic groups: clinical practice, medical research, administration, and non–health care. Group means across gender, age, device type, and department (ie, physician specialty) were considered. Systematic differences in patterns of use across physician demographics and device types were observed.

**Table 3.** Regression results of query types on users’ demographic information.

	(1) Clinical	(2) Research	(3) Administration	(4) Non–health care
Male				
Coefficient	−0.039	−0.009	−0.007	0.055
*P* value	<.001	.006	<.001	<.001
Age				
Coefficient	0.000	0.000	0.000	−0.001
*P* value	.001	.001	<.001	<.001
Department: clinical				
Coefficient	0.051	0.226	0.008	−0.285
*P* value	<.001	<.001	<.001	<.001
Department: medical tech				
Coefficient	0.057	0.132	0.012	−0.201
*P* value	<.001	<.001	<.001	<.001
Mobile				
Coefficient	0.157	−0.333	0.024	0.153
*P* value	<.001	<.001	<.001	<.001
Constant				
Coefficient	0.084	0.442	0.009	0.465
*P* value	<.001	<.001	<.001	<.001
Observations	97,644	97,644	97,644	97,644
*R* ^2^	0.031	0.073	0.005	0.071

### Data Privacy

We examined potential privacy issues arising from the use of GenAI models by physicians. As GenAI models typically work as third-party entities, they often caution users against inputting sensitive information into the system. However, a thorough analysis is essential to determine the extent to which physicians adhere to these guidelines and prevent inadvertent input of sensitive data.

According to the Health Insurance Portability and Accountability Act Privacy Rule, there are 18 types of identifiers that may contain sensitive or personally identifiable information [15]. As users were from China and had limited access to cutting-edge GenAI (Table 4), 15 types of identifiers were detected in our data.

**Table 4.** Percentage of Health Insurance Portability and Accountability Act identifiers in physician queries.^a^

Identifier	Values, n (%)
Human name	958 (0.9)
Location	574 (0.5)
Account name	460 (0.4)
Age	284 (0.3)
Date	231 (0.2)
URL	132 (0.1)
Email address	60 (0.1)
Zip code	59 (0.1)
IP address	39 (0)
Phone number	27 (0)
Body information	21 (0)
Medical number	13 (0)
Vehicle number	1 (0)
Credit card	1 (0)
Fax number	1 (0)

a Fifteen of 18 Health Insurance Portability and Accountability Act identifiers were detected in physician queries. The names of the 15 identifiers are provided. The count and percentage of queries that contained each identifier were recorded. Percentage = count/total number of queries × 100%.

We observed that users disclosed sensitive information on the GenAI platform extremely rarely. Among those rare instances, the most commonly disclosed sensitive information included human names, locations, and usernames, followed by ages, dates, and URLs. We further investigated the distribution of all types of identifiers across the 15 topic categories defined in Table 1. The majority of sensitive information was primarily derived from writing and editing. For instance, 576 of 958 instances of sensitive information on human names were linked to writing and editing, while 302 of 574 location-related sensitive information instances were connected to writing and editing. This suggests that users are less mindful of sensitive information when writing or editing documents. Moreover, sensitive information related to account names (435/460) and URLs (83/132) mainly arose from data analysis and software usage. This indicates a tendency for physicians to disclose sensitive information when copying directories or web sources.

To investigate how the privacy risk varied by user group, we regressed the indicator of whether to disclose sensitive information in the query on users’ demographic information. The results are shown in Table 5. In the first column, we used the ordinary least squares estimator, and in the second column, we used the logit estimator. Their results were consistent. We found that male physicians, compared with female physicians, were more likely to disclose sensitive information. Physicians from the medical technology department faced higher privacy risks than those from the clinical department and other departments. If physicians used the mobile version of the model, their likelihood of posting sensitive information was higher.

**Table 5.** Regression results of privacy disclosure on users’ demographic information.

	(1) Ordinary least squares	(2) Logit
Male		
Coefficient	0.007	0.372
*P* value	<.001	<.001
Age		
Coefficient	−0.000	−0.003
*P* value	.01	.11
Department: clinical		
Coefficient	0.004	0.257
*P* value	<.001	<.001
Department: medical tech		
Coefficient	0.007	0.362
*P* value	<.001	<.001
Mobile		
Coefficient	0.002	0.129
*P* value	.01	.08
Constant		
Coefficient	0.012	−4.370
*P* value	<.001	<.001
Observations	97,644	97,644
*R* ^2^	0.001	—^a^

a Not applicable.

## Discussion

### Principal Findings

This paper leverages a comprehensive query-and-answer dataset to understand how physicians use the current third-party GenAI models. Our findings offer key insights into GenAI model usage and potential in health care. We identify that, when used by physicians, GenAI models are predominantly used for medical research tasks rather than direct clinical applications. This trend highlights a gap between the potential and actual uses of GenAI models in health care, particularly in clinical practice. Health institutions can begin by promoting GenAI use in research applications, such as documentation, literature review, or patient education. These uses can build trust among physicians and demonstrate early value. Once this foundation is in place, institutions should introduce formal validation frameworks, standardized performance benchmarks, and clinical guidelines to assess GenAI for higher-stakes, decision-critical use.

Another observation is the gradual increase in physicians’ reliance on GenAI, starting with informal or personal queries and then evolving to more professional queries. This progression indicates a growing trust and familiarity with the technology. Accordingly, with time and improved integration, GenAI models could become a more requisite tool in health care.

Further, the varied usage patterns based on demographic factors also reveal how different groups within the medical community are adapting to GenAI. These differences not only point to the existence of digital divides but also offer valuable insights into how user needs and behaviors vary by gender, age, device type, and departmental affiliation. First, we showed that female physicians ask a larger proportion of clinical questions and administration questions than male physicians. This suggests that female physicians are more likely to see GenAI as a practical assistant for managing clinical decision-making and routine administrative tasks. Second, we found that younger physicians depend on GenAI in clinical settings more than senior physicians do. Practically, younger physicians’ greater focus on clinical use may reflect higher openness to technology or a need for support with real-time decision-making, while senior physicians’ stronger interest in research may point to their roles in academic medicine. Third, our results showed that physicians using GenAI on computers tend to ask more research-related questions, while those using mobile devices focus more on clinical inquiries. Such device-based variation highlights the importance of context-aware interface design—mobile platforms may benefit from more streamlined, clinically oriented features. Last, we found that staff from clinical departments ask the most research questions, and those from medical technology departments ask the most clinical questions. This indicates that GenAI may be serving as a bridge between technical roles and clinical relevance, helping nonphysician staff understand how their work impacts patient care.

This study also quantifies privacy risks associated with GenAI, an area that has garnered increasing attention. Our findings indicate that while concerns about data privacy are acknowledged, the actual likelihood of sensitive information disclosure is relatively low. Importantly, we identify specific use scenarios—such as drafting or editing documents—where the risk of inadvertent disclosure is higher. These insights underscore the need for clear guidelines and safeguards to ensure responsible use of GenAI in medical contexts. Institutions should address this by establishing clear usage policies, input restrictions, and built-in safety mechanisms. For instance, institutions can enable input filters or redaction suggestions that flag or block sensitive content in real time, particularly for drafting or editing documents.

In sum, this real-world behavioral dataset offers a foundation for evidence-based governance of GenAI in health care. Regulatory agencies and professional bodies can use these insights to craft balanced guidelines that support innovation while safeguarding patient data and care quality.

### Limitations

Although the results of this study are promising, there are a number of limitations that should be considered. First, the majority of participating physicians were based in China, which may limit the generalizability of the results to other health care systems. The health care infrastructure, digital health policies, and physician workflows in China differ significantly from those in other countries such as the United States or those in Europe. For example, in China, a notable proportion of GenAI use cases involves language translation—an application that may be less prevalent among physicians in English-speaking countries such as the United States. However, it is also important to highlight that many aspects of physician workflows are shared globally. Regardless of location, physicians commonly engage in both clinical decision-making and medical research, suggesting that core use cases for GenAI—such as writing and editing, basic medical science, and clinical medicine—are likely to be relevant across health care systems. Second, while this study uses a large-scale quantitative dataset, it does not incorporate qualitative data such as in-depth interviews or open-ended survey responses. Future studies could benefit from integrating qualitative methods to gain richer insights into physicians’ motivations, concerns, and contextual factors shaping GenAI usage. This mixed methods approach could also help explore nuances in trust-building and perceived risks that are difficult to capture through quantitative analysis alone. Last, the data used in this study were observational and derived from real-world usage logs, which means we cannot fully account for physicians’ intentions or the broader clinical context in which queries were made. This could limit the interpretability of some behavioral patterns.

### Conclusions

This study reveals that physicians primarily use GenAI for research rather than clinical tasks, with usage patterns evolving alongside growing trust. Demographic differences shape adoption, and privacy risks remain low but nonnegligible. These findings provide insights into developing safe, effective integration of GenAI into health care practice.

## Supplementary material

10.2196/76941 Multimedia Appendix 1 Supplemental materials for model specification, topic classification, and sensitivity detection.

## Abbreviations

GenAI: generative artificial intelligence
LLM: large language model
