# Supplementary material for: Physician Use of Large Language Models: A Quantitative Study Based on Large-Scale Query-Level Data
Source: J Med Internet Res. 2025 Aug 25;27:e76941. doi: 10.2196/76941 (PMC12377787; doi:10.2196/76941)
Supplement: Multimedia Appendix 1 [file jmir-v27-e76941-s001.docx]

**Supplementary Information Text**

**Model Specification**

The piecewise regression was performed based on Equation [1].

$1\left( QueryType \right)_{ijt}=\beta_{0}{Percentile}_{ijt}+\beta_{1}\left( {Percentile}_{ijt}-15\% \right)1\left( {Percentile}_{ijt}\geq15\% \right)+{\beta_{2}X}_{ijt}+\eta_{i}+\xi_{t}+\varepsilon_{ijt}$[1]

Where $1\left( QueryType \right)_{ijt}$ is an indicator variable representing the category of the query j inputted by user i at time t, which could be $1\left( Clinical \right)_{ijt}$, $1\left( Research \right)_{ijt}$, $1\left( Admin \right)_{ijt}$, or $1\left( Non-healthcare \right)_{ijt}$. These indicators take the value of one if the query j inputted by user i at time t is for clinical practice, medical research, administration, or non-healthcare task respectively and zero otherwise. Here ${Percentile}_{ijt}$represents the percentile of query j in user i’s entire query sequence. A higher value of ${Percentile}_{ijt}$ indicates a prompt occurring later in the sequence, while a lower value indicates an earlier prompt. $X_{ijt}$ represents control variables including the user i’s method of accessing the platform (i.e., via mobile or website) and the model version utilized (i.e., the advanced GPT model or other models) for their query j at time t. Furthermore, $\eta_{i}$ represents user fixed effect, $\xi_{t}$reflects day-of-week fixed effect, and εijt denotes the error term. The coefficient of interest is β0. During the initial 15% of each user’s query sequence, the indicator variable $1\left( {Percentile}_{ijt}\geq15\% \right)$ equals zero. The coefficient β0 captures the change in the probability of query j falling into a specific category during this initial phase. After the first 15% of queries, the indicator variable

$1\left( {Percentile}_{ijt}\geq15\% \right)$ equals one. The first two terms become ${{(\beta}_{0}+\beta_{1})*Percentile}_{ijt}-\beta_{1}*15\%$. Therefore, ${(\beta}_{0}+\beta_{1})$ measures how the probability of query j falling into a particular category change after the initial 15% of the query sequence. We opted for the 15% threshold, as it yields the lowest Akaike Information Criterion (AIC) among different breakpoint options in the piecewise regression.

The results estimated by Equation [1] confirm that there is a “warm-up” period in the usage dynamics, constituting approximately 15% of the initial prompts. After this period, users are less likely to ask administrative or non-healthcare questions and instead consistently focus on professional inquiries.

**Topic Classification**

Large language models (LLMs) such as OpenAI’s ChatGPT and Google’s Genimi represent the forefront of AI tools for various sophisticated tasks [1, 2]. Classification stands out as a significant function of these models [3]. With guidance and demonstrated examples, ChatGPT can be an efficient data annotator [4]. For sentiment analyze, few-shot learning can rival state-of-the-art supervised

methods [5]. ChatGPT is also reported to outperform crowd-workers on classifying tweets dataset [6]. Inspired by these findings, we have opted to implement the GPT-4 model for query classification in our research.

The query data was processed before classification. We first removed anomalous data entries with invalid user IDs and then addressed instances where responses contained “NAN” due to ChatGPT-4’s instability by re-prompting ChatGPT-4 to regenerate responses to the same questions. A total of 106,942 query-and-answer (Q&A) pairs remained. Furthermore, we categorized these Q&A pairs into distinct topic categories through the following comprehensive and iterative process. Initially, we invited a physician from a top-tier hospital in China to manually review the data. This physician suggested a set of 15 categories within four broad groups:

1. ***Medical research*** group contains queries related to

- writing and editing (e.g., proofreading healthcare articles),
- translation (e.g., translating literature from English into the local language),
- medical experimental design (e.g, the steps of a medical experiment),
- data analysis and software use (e.g., debugging for healthcare problems);

1. ***Clinical group*** contains queries related to

- basic medical science (e.g., biological principles of a disease),
- clinical medicine (e.g., disease diagnose),
- medication (e.g., prescription and dose),
- surgical techniques (i.e., surgical procedures);

1. ***Hospital administration*** contains the queries unrelated to - hospital governance (e.g., hospital guideline),

- public education (e.g., writing education blog related to a disease);

1. ***Non-healthcare group*** contains the remaining queries unrelated to

- non-healthcare writing and editing (e.g., writing a non-healthcare speech script)
- non-healthcare data analysis and software use (e.g., debugging for non-healthcare problems),
- non-healthcare entertainment (e.g., writing poetry),
- non-healthcare relationship (e.g., consulting family relationship),
- non-healthcare other (e.g., casual chatting).


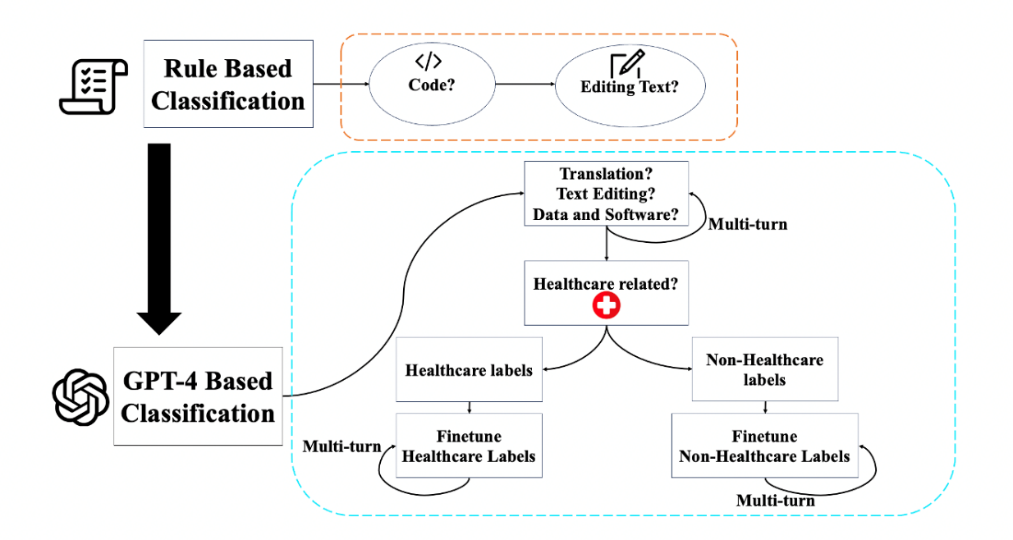


Figure A. Overview of Topic Classification

**
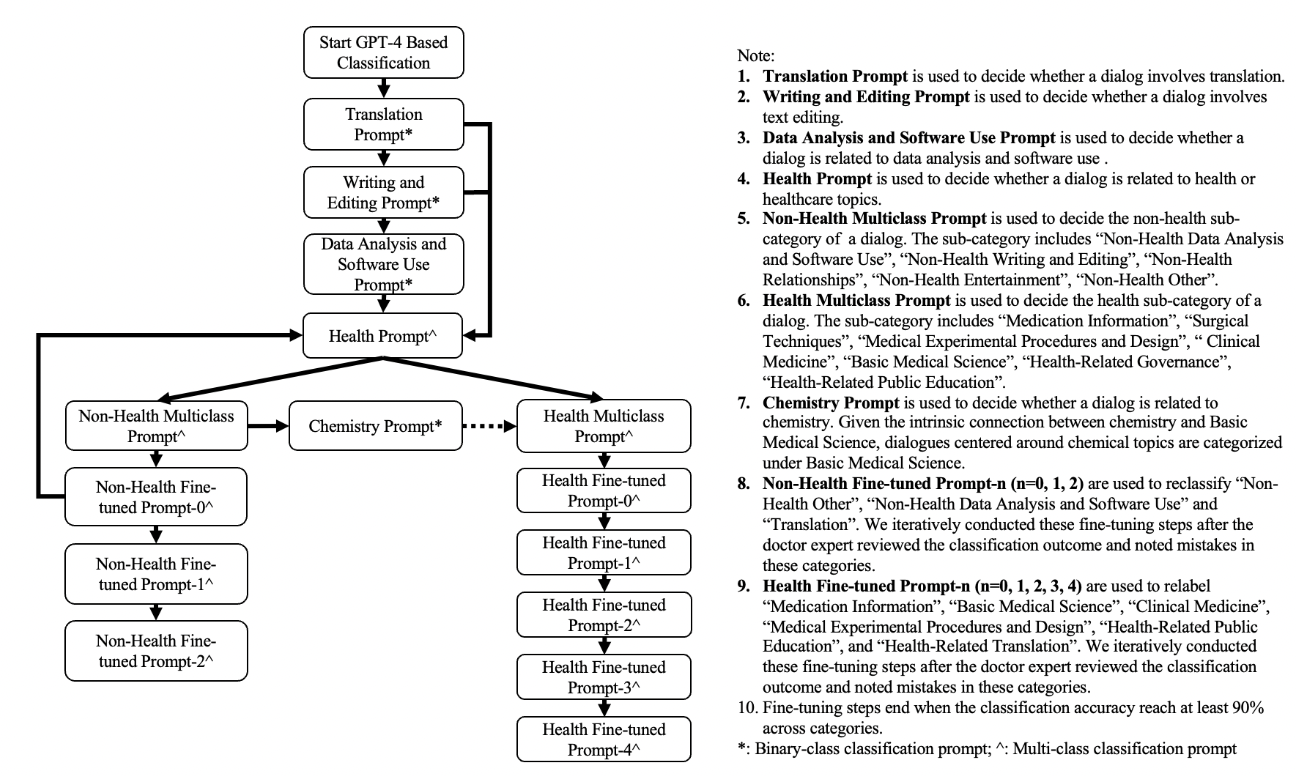
**

Figure B. Details of Topic Classification

Beginning with these predefined categories, we categorized our Q&A pairs into distinct topic categories through the comprehensive and iterative process as shown in Figure A. This classification approach integrated a ChatGPT4-based classifier with a rule-based method. This hybrid approach ensured comprehensive coverage and accuracy in categorizing diverse data points.

First, we employed predetermined rules designed to identify keywords within queries, thereby signaling specific categories. For instance, queries containing markdown code syntax were classified under “Data Analysis and Software Usage,” while those commencing or concluding with terms such as “proofread” or “polish,” and exceeding 100 words in length, were labeled as either “Healthcare Writing and Editing” or “Non-Healthcare Text Writing and Editing.”

Second, ChatGPT-4 is used to assess whether a given content pertained to medical topics. Accomplishing this task in a single run is challenging for ChatGPT-4, even with a substantial number of examples and carefully designed prompts. Prior studies suggest that dividing the task into smaller and more manageable components can enhance its overall performance [7, 8]. Building upon this idea, we decomposed our classification task into smaller and easier subtasks, including binary-class classification and multi-class classification. This decomposition followed a decision-tree-like structure. This structure can be used to break down the classification task into more manageable segments [9], particularly by segmenting the classification into a hierarchy of decision points, as detailed in Figure B.

Specifically, our classification procedure can be viewed as a decision tree, with each node in the tree representing a decision point. At each node, we either prompt the ChatGPT-4 model to determine whether the given query is related to a specific topic (i.e., binary-class classification) or ask it to classify the query into a small set of labels (i.e., multi-class classification). In the binary-class classification, we constrain ChatGPT-4’s responses to either a single word, “YES” or “NO”. In the multi-class classification, we meticulously formulate a definition for each category, which, when merged with the query, creates an integrated prompt used to guide ChatGPT-4 in selecting the most fitting category. We began the tree-structured classification by prompting ChatGPT-4 to identify if the query related to “Translation,” “Writing and Editing,” “Data Analysis and Software Use,” or “Other.” We then attempted to further split each of these four tasks based on whether or not they are related to healthcare. Interestingly, we note that all queries categorized as “Translation” were dominantly determined to be related to healthcare. We next took the remaining categories into account to complete the classification. Note that during the classification process, we identified a new category consisting of chemistry-related queries. We merged them into either the “Basic Medical Science” or “Medication Information” categories because these queries are primarily associated with biology or drugs.

Further, to improve the effectiveness of classification via ChatGPT-4, we took the following steps:

***Step One.*** we implemented few-shot learning approach, a technique that leverages a pre-trained model’s ability to rapidly adapt to new data categories with minimal examples [5, 10, 11]. By providing the ChatGPT-4 model with only a few examples from each category, we generalized it and enabled it to make accurate predictions across all categories.

***Step Two.*** We conducted normalization, which helps to improve classification accuracy. We consolidated multiple spaces into one, converting newline characters into spaces, and capping the length of each query. We also determined the language of queries, shortening a Chinese query to 225 characters due to their usually shorter sentences and shortening an English query to 300 characters due to their comparatively longer sentences. This set of normalization streamlines the classification process by shortening the prompts and ensures a neater prompt structure.

***Step three.*** Context information are often considered in analyzing natural language data. In our framework, the interpretation of the current query can depend significantly on the context of previous interactions. For instance, a user might give instructions for translation in an earlier message and then provide the actual content to be translated in a subsequent one. By sequentially inputting both queries and answers for classification, our model can gain contextual understanding, as these answers are designed to retain information mentioned earlier in the conversation. Consequently, our classification results consistently demonstrate effectiveness, irrespective of whether context information is explicitly provided.

After the classification procedure, we invited our collaborating physician to evaluate the results and provide their feedback. Particularly, we randomly selected 1000 queries for physician review.1 The physician returned feedback regarding the instances of misclassified queries, potential misclassification reasons and category-level accuracy. We took the feedback into account and adjusted our classification pipeline and prompts accordingly. This step is crucial, allowing us to fine-tune the classification process iteratively until we achieved a high category-level accuracy. Category-level accuracy in the last round of physician review showed that both precision and recall reached a minimum of 90% across all categories. The statistics of performance (i.e., the number of correctly predicted instances pertaining to a topic category divided by the total number of instances pertaining to that category) are reported in Table A.

Table A. The Statistics of Performance for Topic Classification

| Label | Precision | Recall |
| --- | --- | --- |
| writing and editing | 0.996 | 0.983 |
| translation | 1 | 0.991 |
| medical experimental design | 1 | 1 |
| data analysis and software use | 1 | 0.966 |
| basic medical science | 1 | 0.977 |
| clinical medicine | 1 | 1 |
| medication | 1 | 1 |
| surgical techniques | 1 | 0.900 |
| hospital governance | 1 | 0.950 |
| public education | 1 | 0.900 |
| non-healthcare writing and editing | 0.924 | 0.994 |
| non-healthcare data analysis and software use | 1 | 1 |
| non-healthcare entertainment | 1 | 1 |
| non-healthcare relationship | 1 | 1 |
| non-healthcare other | 1 | 1 |

1. **Sensitivity Detection**

Health information becomes sensitive when it is individually identifiable [12]. Ac-

cording to the Health Insurance Portability and Accountability Act (HIPAA) Privacy Rule, health information is considered de-identified and insensitive if it neither identifies an individual nor provides a reasonable basis that can lead to identifying an individual.2 The U.S. Department of Health and Human Services offers guidance for the de-identification of protected health information in compliance with HIPAA. The guidance recommends the safe harbor method, which involves the removal of 18 specific types of identifiers. Since the collaborating platform on which we conducted our study focuses on textual prompting, we adjusted the list of sensitive information by excluding non-textual categories, such as fingerprints and voiceprints. The updated list of sensitive information includes 15 types of identifiers: human names, locations, user account

names, ages, dates, URLs, email addresses, zip codes, IP addresses, phone numbers, body information, medical numbers, vehicle numbers, credit cards, fax numbers. In this paper, we examined whether these types of sensitive information are present in physician-submitted queries.

To identify if a given query inputted by a physician contains sensitive information, we adopted the combined methods consisting of regular expression, Presidio [13] and ChatGPT-based detection. Regular expression and Presidio are conventional methods in detecting sensitivity to effectively identify queries that may contain sensitive information [14]. ChatGPT, as a cutting-edge model, is able to further scrutinize the complex sensitive queries that cannot be identified by regular expressions and Presidio. We note that both queries and answers are included in the detection of sensitive information, as answers can provide additional contextual information that enhances detection accuracy.

Regular expression, formulated as patterns to match character combinations in strings, has served as the dominant workhorse of information extraction [14]. A query matching a particular type of patterns is considered containing this type of sensitive information. The patterns used to detect the categories of sensitive information are partly derived from the well-established regular expressions of Presidio. For the patterns that are not predefined, we created custom definitions.

However, regular expressions alone are only capable of recognizing structured content and are insufficient for detecting complex sensitivity. Due to the unstructured nature of our queries, we leveraged more advanced functions of Presidio developed by Microsoft to further detect sensitive information. In particular, Presidio leverages Named Entity Recognition (NER) to construct predefined or custom Personally Identifiable Information (PII) recognizers. NER uses machine learning models pre-trained on large datasets to understand semantic information, thereby improving the identification of sensitive information. In this paper, we utilized the pre-trained models “zh core web lg” and “en core web lg” from the spaCy library [15]. These models effectively detect sensitive entities in Chinese and English, respectively. Note that as human names and locations are less-structured, we mainly used Presidio, rather than regular expressions, to detect them.

Next, in the process of ChatGPT-4 scrutiny, we applied the following two additional techniques. First, we observed that many of the potentially sensitive queries identified by the earlier steps (i.e., regular expressions and Presidio) were often false positives. Many of these queries didn’t actually disclose any sensitive information. For instance, the text “The result is 13300710000” was mistakenly flagged because “13300710000” seems to match the format of a phone number. To improve our detection accuracy, we further employed a few-shot learning approach [2] based on ChatGPT-4 to do fine-tuning. Specifically, we provided a set of Q&A examples related to sensitive information, significantly reducing the false positive rate and enhanced overall performance of sensitivity detection.

Second, to fully leverage ChatGPT-4, we decompose the sensitivity detection task into several sub-tasks, akin to the approach used for of Topic Classification. We asked ChatGPT-4 to determine if a candidate query actually contains sensitive information, which is required to answer “YES” or “NO”. For queries labeled “YES”, we proceed with two additional checks. One check involves specifying the types of sensitivity associated with this query by asking ChatGPT-4 about reasoning process. For example, given the query “Do you know Dr. Jack Lee in AAA Hospital?” and asking for a reasoning process, ChatGPT-4 would indicate that this query is sensitive because it includes a person’s name and location information. Therefore, we can identify that the human name and location are two types of sensitivity linked to this query. The other check is to refine whether the detected sensitive query is factual or fictional, and whether it pertains to common sense or publicly known information.

With this ensemble method, we could accurately detect the sensitivity linked to queries on the collaborating platform. After completing the sensitivity detection, we randomly select 2000 query-answer pairs and recruit a research assistant with text mining experience to manually review the sensitivity detection performance. Although 15 types of sensitive information are detected in the full dataset, only the most frequent six sensitivity categories (i.e., human names, locations, user account names, ages, dates, URLs) were identified in this 2000-instance review sample. The rest categories are not listed due to their infrequency. For the identified categories, the detection performance is reported in Table B. We observe that all precision scores exceed 85 percent, and all recall scores exceed 73 percent across all sensitivity levels.

Table B. The Statistics of Performance for Topic Classification

| Sensitivity Labels | Precision | Recall |
| --- | --- | --- |
| human names | 0.889 | 0.941 |
| locations | 0.917 | 0.733 |
| dates | 1 | 0.75 |
| user account names | 0.857 | 1 |
| ages | 1 | 1 |
| URLs | 1 | 1 |

**Reference**

1. Chang Y, Wang X, Wang J, Wu Y, Yang L, Zhu K, et al. A survey on evaluation of large language models. ACM Trans Intell Syst Technol. 2024;15(3):1-45. [[CrossRef](https://dl.acm.org/doi/full/10.1145/3641289)]
2. Kim G, Baldi P, McAleer S. Language models can solve computer tasks. Adv Neural Inf Process Syst. 2023;36:39648-77. [[CrossRef](https://dl.acm.org/doi/10.5555/3666122.3667845)]
3. Clavié A, Ciceu A, Naylor F, Soulié G, Brightwell T. Large language models in the workplace: a case study on prompt engineering for job type classification. In: International Conference on Applications of Natural Language to Information Systems. 2023. p. 3-17. [[CrossRef](https://dl.acm.org/doi/10.1007/978-3-031-35320-8_1)]
4. He X, Lin Z, Gong Y, Jin A, Zhang H, Lin C, et al. Annollm: making large language models to be better crowdsourced annotators. arXiv [Preprint]. 2023. arXiv:2303.16854. [[CrossRef](https://aclanthology.org/2024.naacl-industry.15/)]
5. Wang Z, Xie Q, Feng Y, Ding Z, Yang Z, Xia R. Is ChatGPT a good sentiment analyzer? A preliminary study. arXiv [Preprint]. 2023. [[CrossRef](https://arxiv.org/html/2304.04339v2)]
6. Gilardi F, Alizadeh M, Kubli M. ChatGPT outperforms crowd workers for text-annotation tasks. Proc Natl Acad Sci U S A. 2023;120(30):e2305016120. [[CrossRef](https://www.pnas.org/doi/10.1073/pnas.2305016120)]
7. Wei J, Wang X, Schuurmans D, Bosma M, Xia F, Chi E, et al. Chain-of-thought prompting elicits reasoning in large language models. Adv Neural Inf Process Syst. 2022;35:24824-37. [[CrossRef](https://dl.acm.org/doi/10.5555/3600270.3602070)]
8. Yao S, Yu D, Zhao J, Shafran I, Griffiths T, Cao Y, et al. Tree of thoughts: deliberate problem solving with large language models. Adv Neural Inf Process Syst. 2024;36. [[CrossRef](https://dl.acm.org/doi/abs/10.5555/3666122.3666639)]
9. Azar T, El-Metwally SM. Decision tree classifiers for automated medical diagnosis. Neural Comput Appl. 2013;23:2387-403. [[CrossRef](https://link.springer.com/article/10.1007/s00521-012-1196-7)]
10. Vinyals O, Blundell C, Lillicrap T, Wierstra D. Matching networks for one shot learning. Adv Neural Inf Process Syst. 2016;29. [[CrossRef](https://proceedings.neurips.cc/paper/6385-matching-networks-for-one-shot-learning.pdf)]
11. Snell J, Swersky K, Zemel R. Prototypical networks for few-shot learning. Adv Neural Inf Process Syst. 2017;30. [[CrossRef](https://dl.acm.org/doi/10.5555/3294996.3295163)]
12. Neamatullah I, Douglass MM, Lehman LWH, Reisner A, Villarroel M, Long WJ, et al. Automated de-identification of free-text medical records. BMC Med Inform Decis Mak. 2008;8:1-17. [[CrossRef](https://bmcmedinformdecismak.biomedcentral.com/articles/10.1186/1472-6947-8-32)] [Medline: [18652655](https://pubmed.ncbi.nlm.nih.gov/18652655/)]
13. Presidio: Data Protection and De-identification SDK. URL: https://microsoft.github.io/presidio/
14. Y. Li, R. Krishnamurthy, S. Raghavan, S. Vaithyanathan, and H. V. Jagadish. Regular expression learning for information extraction. Proceedings of the 2008 conference on empirical methods in natural language processing, pages 21-30, 2008. [[CrossRef](https://dl.acm.org/doi/10.5555/1613715.1613719)]
15. Spacy introduction. URL: https://spacy.io/
